# Cocaine Induces Inflammatory Gut Milieu by Compromising the Mucosal Barrier Integrity and Altering the Gut Microbiota Colonization

**DOI:** 10.1038/s41598-019-48428-2

**Published:** 2019-08-21

**Authors:** Ernest T. Chivero, Rizwan Ahmad, Annadurai Thangaraj, Palsamy Periyasamy, Balawant Kumar, Elisa Kroeger, Dan Feng, Ming-Lei Guo, Sabita Roy, Punita Dhawan, Amar B. Singh, Shilpa Buch

**Affiliations:** 1 Department of Pharmacology and Experimental Neuroscience, University of Nebraska Medical Center, Omaha, NE 68198 USA; 2 Department of Biochemistry and Molecular Biology, University of Nebraska Medical Center, Omaha, NE 68198 USA; 3 VA Nebraska Western Iowa Health Care System, Omaha, NE 68105 USA; 4 Department of Surgery, University of Miami, Florida, FL 33136 USA

**Keywords:** Mucosal immunology, Microbiome

## Abstract

Cocaine use disorder (CUD), a major health crisis, has traditionally been considered a complication of the CNS; however, it is also closely associated with malnourishment and deteriorating gut health. In light of emerging studies on the potential role of gut microbiota in neurological disorders, we sought to understand the causal association between CUD and gut dysbiosis. Using a comprehensive approach, we confirmed that cocaine administration in mice resulted in alterations of the gut microbiota. Furthermore, cocaine-mediated gut dysbiosis was associated with upregulation of proinflammatory mediators including NF-κB and IL-1β. *In vivo* and *in vitro* analyses confirmed that cocaine altered gut-barrier composition of the tight junction proteins while also impairing epithelial permeability by potentially involving the MAPK/ERK1/2 signaling. Taken together, our findings unravel a causal link between CUD, gut-barrier dysfunction and dysbiosis and set a stage for future development of supplemental strategies for the management of CUD-associated gut complications.

## Introduction

Cocaine use disorder (CUD) has traditionally been considered a brain disorder in which alterations of the dopamine neurotransmitter system play a significant role in its psychoactive and addictive effects^1^. Recent studies have demonstrated that cocaine abuse is also related to changes in other neurotransmitters including serotonin, gamma-aminobutyric acid (GABA), norepinephrine, and glutamate^2^. Based on the premise that the limbic system of the brain comprising of a set of interconnected regions regulating pleasure and motivation, is the primary site of action of cocaine, helps explain its high potential for addiction and relapse^1^. Consequently, up to 22.5 million people worldwide are estimated to be affected by CUD, making it a major public health crisis with a high economic and social burden^3^.

In addition to its addictive properties, chronic cocaine use is also associated with weight loss, malnourishment, lack of appetite and reduced blood flow to the gastrointestinal tract^4–6^, suggesting thereby that cocaine also induces damage to the gastrointestinal (GI) tract. These studies suggest that cocaine could dysregulate the expression of tight junction proteins such as the claudin family of proteins, which are integral components of the intestinal barrier, thereby contributing to epithelial damage and increased permeability. Previous studies also have associated cocaine use with altered gut microbiota in humans with HIV infection^7^. Additionally, in another report, alterations in gut microbiota were shown to enhance sensitivity to cocaine reward and locomotor effects in rodents, thereby suggesting a link between gut microbiota and altered behavioral responses to cocaine^8^. Understanding how cocaine dysregulates the GI milieu is critical given the implications of gut microbiota in modulating addictive behaviors associated with cocaine abuse. In the current study, we sought to examine the mechanism(s) underlying cocaine-mediated alterations in gut homeostasis in mice administered cocaine.

Using the 16S rRNA sequencing of the distal colon fecal matter and excreted droppings of the mice administered cocaine, we confirmed significant alterations of gut microbial colonization, including the suppression of microbiota critical for butyrate synthesis, that is essential for the maintenance of normal gut homeostasis. Furthermore, our findings also identified ERK1/2-dependent gut-barrier dysregulation as a potential mechanism underlying cocaine-induced gut dysbiosis and the associated inflammatory environment. Overall, our findings demonstrate a critical association between CUD and gut-barrier alterations in regulating the gut microbial colonization and inflammatory milieu.

## Results

### Cocaine induces gut dysbiosis

**Figure 1:**
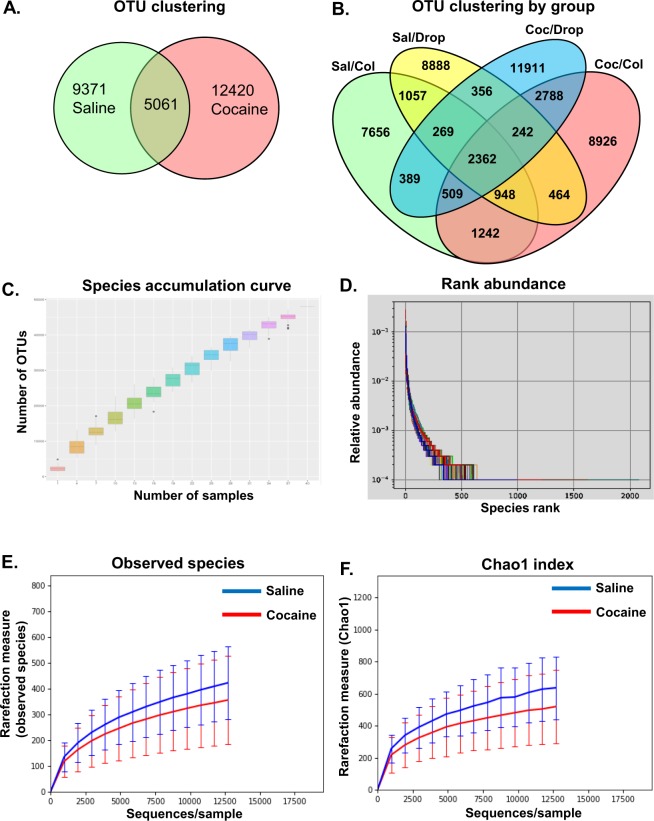
Alpha diversity metrics in cocaine- or saline-administered mice. Wild-type mice (C57BL/6) were administered cocaine (i.p, 20 mg/kg) or saline for seven consecutive days followed by euthanasia within 1 hr of the last injection. Fecal droppings and distal colon fecal matter were collected for 16S-rRNA sequencing. As shown in (**A**,**B**), cocaine administration altered several Operational Taxonomic Units (OTU) both the colon and the fecal droppings. Metrics for species accumulation curve (**C**), rank abundance (**D**), Observed species (**E**) and Chao1 (**F**) phylotype diversity are shown. n = 10/group.

**Figure 2:**
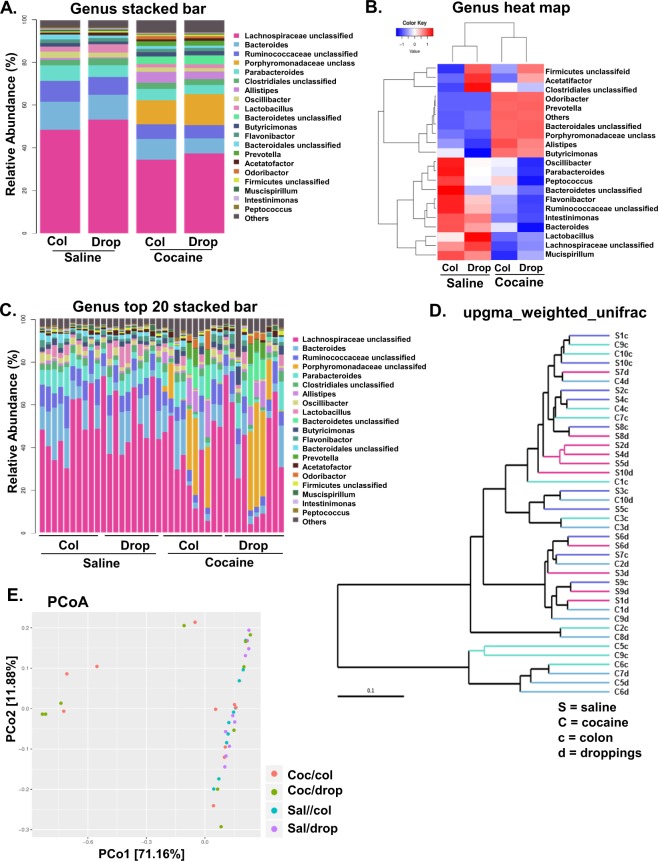
Cocaine-administration in mice induced gut dysbiosis. Wild-type mice (C57BL/6) were administered cocaine (i.p, 20 mg/kg) or saline for seven consecutive days followed by euthanasia within 1 hr of the last injection. Fecal droppings and distal colon fecal matter were collected for 16S-rRNA sequencing. Several bacterial genus OTUs were altered in the presence of cocaine compared with controls as shown by the stacked bar (**A**) and the heat map (**B**) in pooled samples for each group. Cocaine administration affected microbial composition as shown by relative abundance of 16S rRNA transcripts in each individual animal at the genus level (**C**). UPGMA weighted unifrac and principal coordinates analysis (multidimensional scaling, MDS) show the clustering of microbiota from cocaine-administered mice (**D,E** respectively). S = Saline, C = cocaine, c = colon, d = droppings, col = colon, drop = droppings. n = 10/group.

To achieve detailed insight into the causal association of cocaine exposure and gut dysbiosis we first used 16S rRNA sequencing to analyze the microbiota from the fecal droppings and the colon of mice that were administered cocaine (20 mg/kg; i.p./day) for 7 consecutive days. We found 5061 OTUs that were common between the cocaine-exposed and control groups and, 9371 and 12420 OTUs that were specific to the saline controls and cocaine-exposed groups respectively (Fig. 1A). We further clustered the OTUs between the fecal droppings and the colon, and found 2362 OTUs that were common between the fecal droppings and colon irrespective of cocaine- or saline-exposure; and 11911 and 8926 OTUs that were specific for cocaine-exposed fecal droppings and colon, respectively (Fig. 1B). Species accumulation reached saturation (Fig. 1C), richness and evenness (Fig. 1D). Bacterial alpha diversity analyzed as observed species, or the Chao1 index remained unchanged between the cocaine-exposed and the saline administered mice (Fig. e,f). We next analyzed for shifts in beta diversity and observed alterations in bacterial composition based on the weighted UniFrac ANISOM diversity metric (R = 0.592, p = 0.001) thereby suggesting microbiota dissimilarities between cocaine- versus saline-administered mice. To better understand the effect of cocaine on the gut microbial composition, we next studied the effect of cocaine exposure on the taxonomic composition at the genus level. Cocaine exposure decreased colonization in the colon of four genera, i.e., *Mucispirillum*, *Butyricicoccus*, *Pseudoflavonifractor* and unclassified *Ruminococcaceae* with a concomitant increase in colonization of other genera such as *Barnesiella*, and unclassified members of *Porphyromonadaceae*, *Bacteriodales*, and Proteobacteria (Fig. 2A–C, Table 1). Fecal droppings of the cocaine-exposed mice also demonstrated decreased abundance of *Lachnospiracea incertae sedis*, *Pseudoflavonifractor* and *Streptophyta* and increased abundance of *Turicibacter*, *Alistipes*, *Odoribacter* and unclassified members of *Porphyromonadaceae* and Proteobacteria, (Fig. 1A–C, Table 2) thereby suggesting alterations of gut microbial homeostasis. Furthermore, it was also found that bacterial population in the colon and fecal droppings of cocaine-administered mice clustered separately from saline controls, based on the phylogenetic distance UPGMA weighted UniFrac (Fig. 1D) and principal coordinate analysis (Fig. 1E).

**Table 1:** Bacterial genus altered by cocaine in the colon.

Genus	Fold change	P value	Significance	Regulation
*Barnesiella*	10.87	0.0004	yes	up
*Porphyromonadaceae unclassified*	9.10	0.0041	yes	up
*Bacteroidales unclassified*	7.16	0.0015	yes	up
*Clostridiales Family XIII*. *Incertae Sedis unclassified*	1.36	0.0051	yes	up
*Erysipelotrichaceae unclassified*	1.84	0.0065	yes	up
*Streptococcus*	6.71	0.0072	yes	up
*Sphingomonas*	4.42	0.0020	yes	up
*Pasteurellaceae unclassified*	4.36	0.0130	yes	up
*Proteobacteria unclassified*	6.71	0.0166	yes	up
*Mucispirillum*	−1.34	0.0191	yes	down
*Desulfovibrionaceae unclassified*	4.53	0.0272	yes	up
*Alistipes*	2.58	0.0413	yes	up
*Ruminococcaceae unclassified*	−0.51	0.0191	yes	down
*Pseudoflavonifractor*	−0.67	0.0413	yes	down
*Butyricicoccus*	−0.88	0.0588	no	down
*Hydrogenoanaerobacterium*	−1.03	0.0584	no	down

**Table 2:** Bacterial genus altered by cocaine in the fecal droppings.

Genus	Fold change	P value	Significance	Regulation
*Porphyromonadaceae unclassified*	10.74	0.002	yes	up
*Proteobacteria unclassified*	7.18	0.041	yes	up
*Turicibacter*	0.48	0.022	yes	up
*Alistipes*	2.93	0.023	yes	up
*Lachnospiracea incertae sedis*	−1.25	0.041	yes	down
*Odoribacter*	11.68	0.036	yes	up
*Streptophyta*	−3.95	0.036	yes	down
*Pseudoflavonifractor*	−1.07	0.028	yes	down

We next analyzed the effect of cocaine exposure at the species level and found depletion of unclassified species of *Mucispirillum*, *Butyricoccus and Ruminococcaceae* with a concomitant upregulation of unclassified species of *Porphyromonadaceae*, *Bacterroidales*, *Barnisiella*, Proteobacteria and *Alistipes* among others in the colon (Supplementary Table S1). Fecal droppings of cocaine-exposed mice demonstrated a similar dysregulation of microbial homeostasis involving downregulation of several bacterial species including unclassified *Lachnospiracea incertae sedis*, *Streptophyta* and uncultured *Lactobacillus* species (Supplementary Table 2). Cocaine-induced alterations in *Lachnospiracea*, *Butyricoccus*, *Ruminococcaceae* and other butyrate-producing bacteria suggested disturbances in gut energy balance as well as butyrate-mediated neural and immune functions.

### Cocaine administration alters gut homeostasis

**Figure 3:**
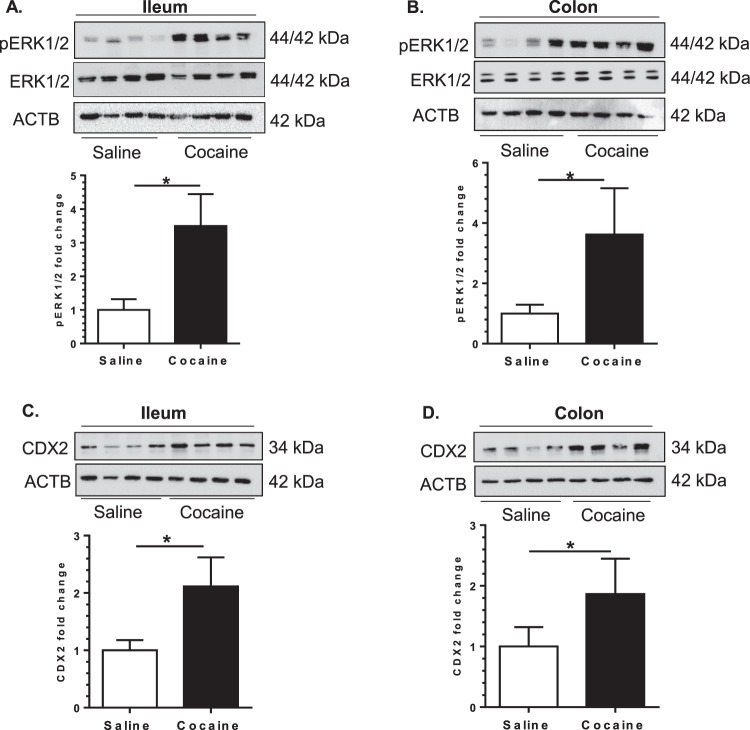
Cocaine administration augments cell survival and metabolic signaling in mice intestine. Mice were administered cocaine (i.p. 20 mg/kg) once per day for 7 days, sacrificed 1 h post the final cocaine injection followed by a collection of ileum and colon for assessing the expression of ERK 1/2, pERK1/2 and CDX2. As shown in a & b, cocaine-administered mice demonstrated upregulated expression of phosphorylated ERK 1/2 kinase and CDX2 in both the ileum and colon (n = 4/group). Western blot quantification is shown under each blot. Blot images are cropped from different membranes used for data collection and analysis (See Supplemental Figures). Data are shown as mean ± SEM and *p < 0.05.

**Figure 4:**
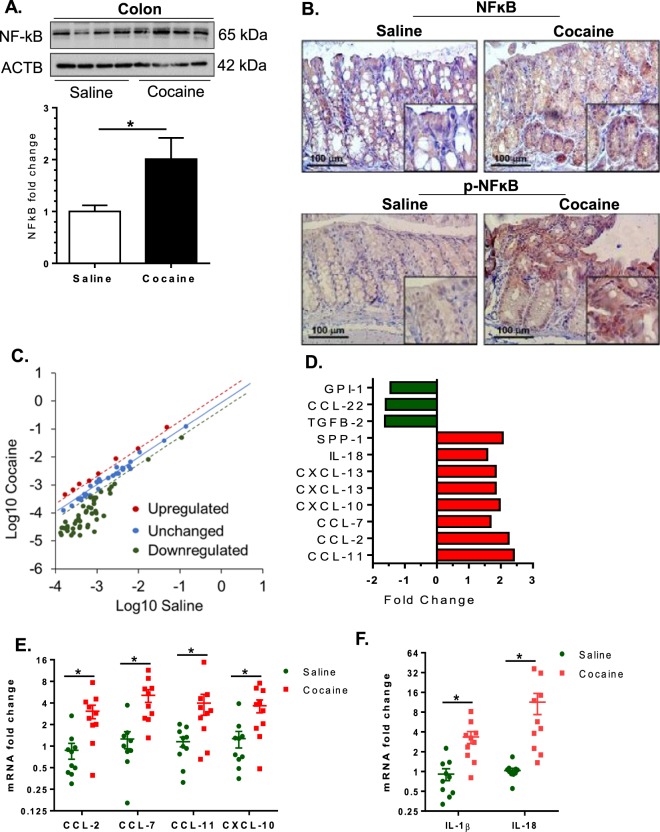
Cocaine administration induced an inflammatory gut milieu. Mice were administered cocaine (i.p. 20 mg/kg) for 7 days, sacrificed 1 h post the final cocaine injection and colon collected for expression analysis of inflammatory markers. (**A**,**B**) Upregulated expression of total NF-ĸB and pNF-ĸB (p65) in the colon. (**C**,**D**) Mouse common cytokines and chemokines PCR array (PAMM 150, Qiagen) analysis showed upregulation of several cytokines and chemokines. Fold change was calculated in cocaine treated mice relative to saline treated controls. (**E**,**F**) q-PCR validation showed significant increases in CCL–2, CCL-7, CCL-11, CXCL-10, IL-1β, and IL-18. Blot images are cropped from different membranes used for data collection and analysis (See Supplemental Figures). (n = 10 /group, *p < 0.05).

Next, we sought to examine whether cocaine exposure could affect gut homeostasis. We first assessed phosphorylation of ERK1/2 kinase that lies upstream of both CDX-2 and NF-κB - transcription factors that are critical for regulating gut homeostasis. While the levels of total ERK1/2 kinase remained unchanged in cocaine-administered mice, its phosphorylation was significantly upregulated in both the colon and ileum of cocaine-exposed mice compared with the control group (Fig. 3A,B). As shown in Fig. 3C,D, there was significant upregulation of CDX2 expression both in the colon and ileum of cocaine administered mice. A concomitant increase in the expression of both total and phosphorylated NF-κB was also observed in the colon of cocaine administered mice (Fig. 4A,B), thereby underscoring the role of cocaine in the modulation of gut homeostasis.

The next step was to determine whether cocaine exposure resulted in the induction of an inflammatory environment in the gut by analyzing the gene expression of specific chemokines and cytokines. Total RNA from the colon was subjected to qPCR analysis using a Qiagen mouse (common) cytokines and chemokines PCR array panel (PAMM 150, Qiagen). As shown in Fig. 4C,D, there was increased expression of several chemokines and cytokines and also downregulation of some others in the colon of cocaine administered mice compared to the control group. Further validation of these findings was also done using qPCR analysis for chemokines such as CCL-2, CCL-7, CXCL-10, CCL-11 as well as for cytokines IL-18 and IL-1β. As shown in Fig. 4E,F, cocaine exposure was found to induce a pro-inflammatory gut milieu. Notably, additional analysis using sera of either saline or cocaine-administered mice further demonstrated increases in known pro-inflammatory mediators such as Lipopolysaccharide Binding protein (LBP) and soluble CD14 ligand (sCD14) (Supplementary Fig. S1).

### Cocaine administration alters mucosal epithelial barrier composition and integrity

**Figure 5:**
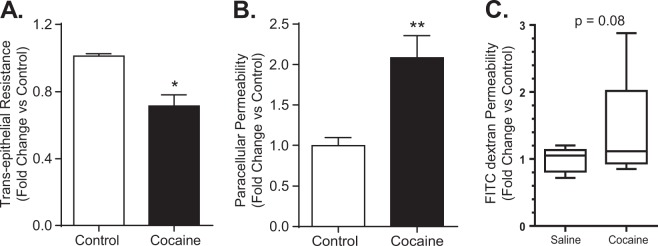
Cocaine exposure inhibited trans-epithelial resistance and increased permeability in Caco-2 cells and in mice. Caco-2 epithelial cells were seeded in transwell chambers and exposed to cocaine (10 µM) or left untreated. (**A**) Exposure of Caco-2 cells to cocaine resulted in decreased trans-epithelial electrical resistance (TEER) and (**B**) increased paracellular permeability as shown by apical-basal pass of FITC-dextran dye (4 kDa). Results were expressed relative to the initial TEER value and presented as mean ± SEM for three independent experiments. *p < 0.05. (**C**) Mice were administered cocaine (20 mg/kg, i.p.) or saline for seven days and orally gavaged with FITC Dextran (4 kDa) on the final day of cocaine injection followed by collection of blood 4 h post FITC dextran gavage. (n = 9/group), p = 0.08.

Gut barrier dysfunction is often attributed to tight junction dysregulation, which includes the claudin family of proteins. Based on the premise that expression of claudins is altered in an inflammatory milieu, we next sought to assess for the barrier integrity by measuring the trans-epithelial electrical resistance (TEER) in the widely used *in vitro* model of intestinal epithelial cells - polarized monolayers of Caco-2 cells^9–11^. Confluent cell monolayers on transwell filter support (0.04 µM pore size) were either exposed to cocaine (10 µM) for 24 h or left unexposed followed by TEER measurements. As shown in Fig. 5A, cocaine exposure resulted in a significant reduction of TEER in Caco-2 cells. Complementary analysis of the paracellular permeability using apicobasal passes of a FITC-dextran dye (4 kDa) in these cells further demonstrated a significant increase in permeability compared with control cells (Fig. 5B).

We next assessed intestinal permeability in mice that were administered cocaine (20 mg/kg, i.p.) or saline (n = 9/group) for seven days and subsequently administered FITC Dextran (4 kDa) on the final day of cocaine injection followed by collection of blood after 4 h for detection of FITC by flourometry. As shown in Fig. 5c, cocaine increased intestinal permeability as FITC-dextran concentration in blood was higher in mice administered with cocaine compared with mice administered with the vehicle (p = 0.08).

**Figure 6:**
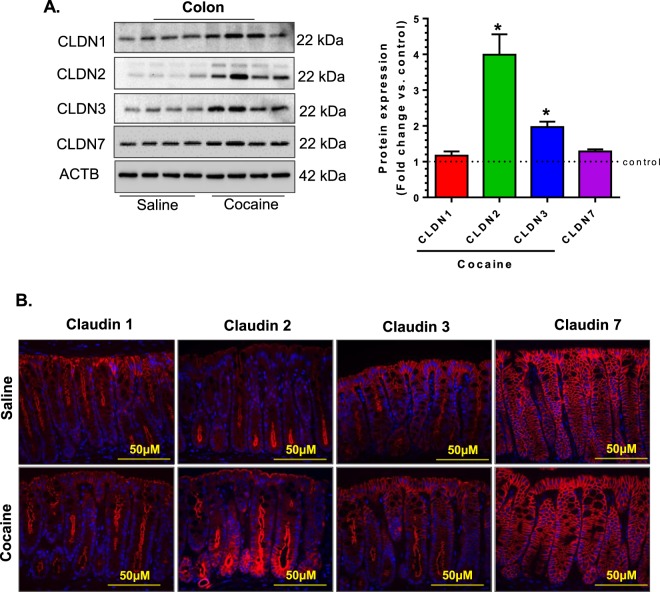
Cocaine-administration modulated the expression of tight junction proteins in the intestinal epithelium by altering claudin protein expression. Mice were administered cocaine (i.p. 20 mg/kg) for 7 days, sacrificed 1 h post the final cocaine injection and colon collected for expression analysis of claudin family of proteins. (**A**) Western blotting of colon tissues for claudin-1, -2, -3 and -7 and (**B**) immunofluoresence staining revealed altered claudin protein expression in the colon. Blot images are cropped from different membranes used for data collection and analysis (See Supplemental Figures). (n = 4group, *p < 0.05).

We next examined the expression and cellular distribution of the important claudin proteins (claudin-1, 2, 3 and 7) in the intestinal epithelium. Interestingly, the expression of these tight junction proteins was found to be upregulated in the colon of cocaine administered versus saline control mice (Fig. 6A). Notably, the expression of the pore-forming claudin- 2 was upregulated the most with up to 4-fold increase compared with controls. Analysis using immunofluorescence staining and imaging further validated alterations in the expression and cellular distribution of these proteins (Fig. 6B).

### Cocaine administration also modulated claudin expression in 3d-culture of colonic crypts potentially by inducing ERK1/2 signaling

**Figure 7:**
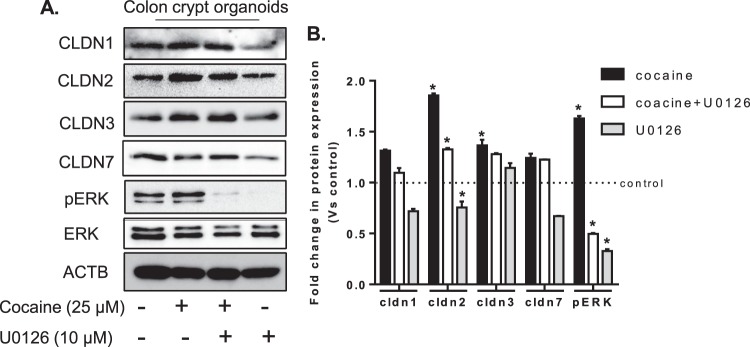
Cocaine-mediated dysregulation of claudins involves activation of ERK 1/2. Colon crypt organoids were exposed to cocaine (25 µM) in the presence or absence of specific inhibitor of MAP/ERK1/2 signalling, U0126 (10 µM). Cocaine exposure modulated the expression of claudins that was differentially attenuated by U0126. Blot images are cropped from different membranes used for data collection and analysis (See Supplemental Figures). Results are presented as mean ± SEM for three independent experiments, *p < 0.05.

We next sought to assess if alterations in the expression of claudin proteins can be recapitulated in 3d- culture of the colon crypt organoids that were either exposed to cocaine or left unexposed in the presence or absence of the ERK1/2 inhibitor U0126. As shown in Fig. 7A, exposure of colon crypt organoids to cocaine modulated expression of claudins and similar to the changes *in vivo* in cocaine-treated mice (versus control), and these changes in claudin expression were accompanied with a significant increase in expression of phosphorylated ERK1/2. There are reports, including ours, demonstrating MAPK/ERK-mediated regulation of claudin expression and localization^12–15^. We thus next sought to explore whether inhibition of ERK phosphorylation could restore cocaine-mediated disruption of claudin expression. To address this, colon crypt organoids were pretreated with the specific inhibitor of MAP/ERK1/2 kinase inhibitor - U0126 (10 µM), followed by exposure of the crypt cultures to cocaine and assessed for the expression of claudins. Pretreatment of crypt organoids with U0126 ameliorated cocaine-mediated expression of claudin especially claudin-2, compared to the control crypt organoids. In the same samples, the cocaine induced claudin-1, −3, & −7 expression was not affected by inhibiting ERK-activation (Fig. 7A,B). Taken together, our data suggested potential role of ERK1/2 signaling in cocaine induced modulation of specific claudin expression and barrier deregulation.

## Discussion

Cocaine use disorder (CUD) is estimated to affect up to 22.5 million people worldwide^3^ resulting in marked changes in behavior and lifestyle emanating from its psychoactive and addictive effects. While most studies focus on the effects of cocaine on the brain, the current study was undertaken to assess the effects of cocaine on dysregulation of gut homeostasis including alterations in microbiota diversity, epithelial barrier function as well as innate immunity. Previous studies have demonstrated that depletion of gut microbiota by antibiotics in mice resulted in enhanced sensitivity to the reward and sensitizing properties of cocaine in the context of cocaine-mediated behavioral plasticity^8^. The current study provides insights into the mechanism(s) of cocaine-induced dysregulation of gut-barrier function, microbial colonization, and inflammation by directly examining the effect of cocaine administered by the intraperitoneal route on the intestinal microbial colonization, in both the fecal droppings and the colon. Most notably, our findings suggest that cocaine administration specifically depletes the *Mucispirillum*, *Ruminococcaceae*, *Lachnospiracea*, *Pseudoflavonifractor* and *Butrycicoccus* bacteria, that are key producers of short-chain fatty acids (SCFA) and related metabolites and that play critical roles in maintaining mucosal epithelial and immune homeostasis. Importantly, SCFA constitute a significant source of energy for the resident microbiota in the colon, and thus by inference depletion of SCFA producers could, in turn, contribute to dysbiosis^16^. In this regard, Kiraly *et al*. demonstrated that supplementation of SCFA reverses cocaine-mediated behavioral effects in antibiotic-treated mice, thereby underscoring the critical role of bacterial metabolites in modulating behavioral changes observed in these animals^8^. Since SCFA are well-known histone deacetylase inhibitors (HDACs)^17^, and also since previous reports have shown that administration of HDAC inhibitors in rodents resulted in reduced behavioral responses to cocaine^18,19^, it is plausible to envision that cocaine-mediated behavioral changes are regulated by a mechanism(s) involving alterations in gut microbiota. Intriguingly, similar to injection of cocaine, a recent study on rats chronically exposed to volatile cocaine also demonstrated similar alterations in the gut microbiota^20^.

Cocaine has been shown to activate NF-κB signaling pathway resulting in upregulated expression of pro-inflammatory cytokines and adhesion molecules in various cell types^21–26^. Furthermore, phosphorylated ERK1/2 that lies upstream of NF-κB is also known to activate CDX-2, a key transcription factor expressed in the gut and that modulates the expression of several genes involved in cellular proliferation, differentiation, and inflammation including integral proteins of the barrier^27–30^. The current study demonstrated that i.p. cocaine administration induced an inflammatory environment in the gut as evidenced by increased expression of multiple pro-inflammatory cytokines (IL-18, IL-1β) and chemokines (CCL-2, CCL-7, CXCL-10, CCL-11) and furthermore, this involved increased activation of the transcription factors NF-κB and CDX-2. Understanding the contribution of cocaine-induced gut inflammation in mediating neuroinflammation and, its role in the development of drug addiction warrants further investigation.

We also observed that cocaine upregulated the expression of claudin-2 in the colon. Claudin-2 has been shown to form paracellular channels that are permeable to cations and water^31–33^. Interestingly, upregulation of claudin-2 has also been shown to increase tight junction permeability to sodium ions and water during enteric pathogen infection^34^. Our data suggest that cocaine increased the expression of claudin-2, which in turn, contributed to increased epithelial permeability. Further studies in models of claudin genetic ablation are required to ascertain the contribution of claudins to cocaine induced barrier dysregulation and microbial translocation.

An intriguing potential mechanism that could provide a plausible explanation underlying cocaine-mediated dysregulation of gut homeostasis was the breach of the gut-barrier integrity, key player involved in regulation of intestinal inflammation. Recent studies have suggested a feedback mechanism between the gut-barrier function and inflammatory cytokines such as IL-13, TNF-α, IL-1β, and IFN-γ^35–39^, thereby implying a positive association between gut-barrier dysregulation, dysbiosis, and inflammation. Our *in vitro* and *in vivo* analyses support such a postulate and demonstrate that cocaine administration could impact the gut-barrier composition and integrity, leading in turn, induction of gut dysbiosis and inflammatory milieu. It is worth noting that deregulation of claudin protein expression and its altered cellular distribution has emerged as a critical factor in the disruption of mucosal barrier integrity^31,34,40,41^. Furthermore, the potential non-canonical role of these proteins in regulating mucosal epithelial and immune homeostasis including Notch-signaling have also been described^42–44^. Our data demonstrating that cocaine-induced alterations in claudin proteins were rescued by inhibition of ERK1/2 signaling further supports the possibility of a non-canonical role and warrants future in-depth investigation.

While our finding suggest that cocaine can disrupt both the microbiota and compromise gut barrier integrity, we cannot conclusively suggest whether cocaine impact on the microbiome leads to permeability changes or that cocaine effects on the gut epithelium lead to altered microbiota. Additional studies using germ-free or antibiotic-treated mice are warranted to help resolve this issue.

In summary, the overall findings herein demonstrate gut homeostasis dysregulation as a severe health concern related to abuse of cocaine and identify gut-barrier dysregulation, altered gut microbiota colonization, and inflammation as contributing factors, potentially acting in an inter-dependent manner. The possibility that these cocaine-mediated changes in gut homeostasis could subsequently contribute to neuroinflammation is highly plausible. Understanding cocaine-induced gastrointestinal tract dysregulation thus appears to be critical in light of the emerging role of the gut in modulating behavior especially the addictive behaviors.

## Materials and Methods

### Reagents

The following antibodies and reagents were purchased from the sources indicated: ERK 1/2 (R&D Cat# 9107S); pERK 1/2 (R&D Cat# 9101S); CDX2 (LSbio Cat# LS-B9317); NFĸB (Abcam cat # 16502), pNFĸB p65 (Cell Signaling Cat # 3031S); Claudin 1 (Invitrogen Cat# 51-9000); Claudin 2 (Invitrogen Cat# 32-5600); Claudin 3 (Invitrogen Cat# 341700); Claudin 7 (Invitrogen Cat# 374800); goat anti-rabbit-HRP (Santa Cruz Biotechnology Cat# sc-2004); goat anti-mouse-HRP (Santa Cruz Biotechnology Cat# sc-2005) and; actin (Sigma-Aldrich Cat# A1978). U0126 (Cat # 19-147) was from Sigma-Aldrich.

### Mice and drug treatments

All animal procedures were performed in strict accordance with the protocols approved by the Institutional Animal Care and Use Committee of the University of Nebraska Medical Centre and the National Institutes of Health. Eight to ten weeks old male mice (C57BL/6N) were purchased from Charles River Laboratories (Wilmington, MA, USA). They were housed under conditions of constant temperature and humidity on a 12-h light, 12-h dark cycle, with lights on at 0700hrs. Food and water were available *ad libitum*. Mice were randomly divided into two groups administered either Cocaine (C5775, Sigma-Aldrich, 20 mg/kg, i.p.) or saline once a day for 7 days with 2 cages per group (n = 5/cage), The cocaine dose of 20 mg/kg, given once daily, has been shown to induce cocaine’s rewarding effects in C57BL/6 mice as reflected by the development of conditioned place preference in these mice^45^. Several studies from our group have utilized this dose over a 7-day period and observed significant molecular changes^46–48^. We thus used a cocaine dose (20 mg/kg; i.p./day) known to produce robust molecular and behavioral changes to examine the relationship between cocaine exposure and operational taxonomic units (OTU) in the gut. Mice were sacrificed by isoflurane anesthesia 1 hour post the final drug injection for gut removal. Droppings and colon fecal matter were collected for microbial DNA isolation. Mice droppings and fecal samples were immediately frozen on dry ice and then stored at −80 °C. Colon or ileum tissues were collected and used for extraction of protein and total RNA. mRNA and protein levels of pro-inflammatory cytokines and signaling proteins were assessed by quantitative RT-PCR and western blotting, respectively.

### DNA isolation, 16S rRNA sequencing, and analysis

Bacterial DNA was extracted from fecal matter using the PowerSoil DNA isolation kit (MO Bio, Carlsbad, CA, USA Cat # 12888-100) according to manufacturer’s protocol. DNA samples were stored at −20 °C (or −80 °C for longer storage) until amplification. Polymerase chain reaction (PCR) of the variable 3 and 4 (V3 and V4) of the 16S rRNA gene was performed using 515F (5′-GTGYCAGCMGCCGCGGTAA-3′) and 806R (5′-GGACTACNVGGGTWTCTAAT-3′) primers^49,50^. Thermocycling conditions were 98 °C for 10 s, 58 °C for 30 s, 72 °C for 45 s for 35 cycles and 72 °C for 10 min. DNA was sequenced using the Illumina MiSeq platform at LC Sciences (Huston, Texas). Briefly, Operational Taxonomic Units (OTUs) were clustered at 97% sequence similarity using cd-hit. Taxonomy was assigned using the Ribosomal Database Project (RDP) classifier v.2.1 against the Greengenes reference database (13_8). Quantitative Insights Into Microbial Ecology (QIIME) was used for subsequent analysis of within- and between- community diversity (alpha and beta diversity). The relationship between 7-days cocaine administration (20 mg/kg) and microbiota was explored using Principal Coordinate Analysis (PCoA) on unweighted UniFrac phylogenetic distances between communities. The dissimilarity distance between the cocaine-exposed mice group and the saline control group was tested using ANOSIM statistical method.

### RNA isolation and quantitative polymerase chain reaction (qPCR) from gut tissue

qPCR was performed as previously described^51^. Total RNA was extracted using Quick-RNA MiniPrep Plus (Zymo Research, R1058) as per the manufacturer’s protocol and quantified using NanoDrop 2000 spectrophotometer. RNA was transcribed into complementary DNA using Verso cDNA kit (Invitrogen, AB-1453/B) according to the manufacturer’s instructions. qPCR was performed by using TaqMan Universal PCR Mastermix (TaqMan, 4324018). The reactions were set up as follows: 10 µl TaqMan Mastermix, 1 µl forward/reverse primers and 7 µl molecular grade water and 2 µl cDNA. The QuantStudio 3 real-time PCR system (Applied Biosystems, Grand Island, NY) was used for program running. Mouse primers for CCL-2 (Mm00441242_m1, cat# 4331182); CCL-7 (Mm00443113_m1, cat# 4331182); CCL-11 (Mm00441238_m1, cat# 4331182); CXCL-10 (Mm00445235_m1, cat# 4331182); IL-1β (Mm00434228_m1, cat# 4331182), IL-18 (Mm00434226_m1, cat# 4331182) and actin (Mm99999915_g1, cat# 4331182) were purchased from ThermoFisher.

### Serum collection and cytokine assays

Blood was collected via cardiac puncture and centrifuged (2000g) for 15 min. The serum was collected and stored at −80 °C. Serum sCD14 (R&D, MC140) and LBP (Abcam, ab213876) were quantified by ELISA according to the manufacturer’s instructions.

### Western blotting

Colon tissues or crypts were lysed in RIPA buffer supplemented with a protease inhibitor cocktail (ThermoFisher Scientific, 78430) followed by ultrasonication for 15 sec, at 80% amplitude. Western blotting was performed as previously described^51^. Lysates were cleared by centrifugation at 12000 *g* (10 min; 4 °C). Using Thermo Scientific BCA kit (Cat# 23225), protein concentration was quantified by the BCA method. Equal amounts of protein (10–20 µg) were electrophoresed in a sodium dodecyl sulfate-polyacrylamide gel under reducing conditions. Following transfer, PVDF membranes (Millipore, IPVH00010) were blocked with 5% nonfat dry milk for 60 mins at room temperature. Next, membranes were probed with primary antibodies overnight at 4 °C, washed with TBS-T, then incubated with appropriate HRP-conjugated secondary antibodies and developed with SuperSignal West Dura or Femto substrate. Densitometric analyses were done using NIH ImageJ software (ImageJ v1.44, NIH). Protein amounts were normalized to β-actin.

### Trans-epithelial electrical resistance (TEER) assay

Caco-2 cells were cultured on polycarbonate transwell filter supports (0.04 µM pore size), exposed to cocaine (10 µM) and trans-epithelial electrical resistance (TEER) was measured as previously described^41,52^. Caco-2 cells form cell monolayers with characteristics of mature enterocytes and have been widely used as an *in vitro* model of intestinal epithelial cells^9–11,53,54^. Results were expressed relative to the initial TEER value and presented as mean plus or minus standard error for three independent experiments.

### FITC dextran permeability flux assay

Caco-2 cells were cultured on transwell filter support to confluency and exposed to cocaine 10 µM) for 24 h. Five microliters of FITC conjugated–dextran (120 mg/ml, 4 kDa, Cat # FD4-1G, Sigma) was added to the apical compartment of the transwell chamber system for 24 hours. The fluorescence intensity in the basal compartment was measured by fluorometry (excitation and emission wavelength of 485 nm and 520 nm, respectively).

### Intestinal permeability

To access *in vivo* intestinal permeability FITC-dextran (120 mg/ml, 4 kDa, Cat # FD4-1G, Sigma) was orally gavaged into mice 4 hour prior to blood collection. After sacrifice, serum FITC-dextran fluorescence intensity was measured by Spectrofluorometry (Bio Tek Instruments).

### *Ex*-*vivo* murine colon crypt organoid culture

Wild type mice (C57Bl6; 6–8 weeks) were sacrificed, colons were removed and flushed with cold PBS to remove the colon contents. Thereafter, the colon was opened longitudinally using a sharp scissor and epithelial layer was separated from the muscles using a razor blade and tweezer. Once separated, the epithelial layer was chopped into small pieces using a new razor blade and colonic crypts were cultured into a Matrigel bedding as for 3D-organoid culture. DMEM/F-12 culture medium supplemented with penicillin/streptomycin, glutamine, N2 and B27 was used to maintain the 3D-crypts cultures. For our studies, cocaine (25 µM) was mixed with the matrigel bedding prior to the culture of the colon crypts. MEK Inhibitor U0126 (10 µm) was used to study ERK1/2 activation upon cocaine treatment. Upon completion of the experiment, colonic crypts were removed from matrigel by using cold 1 mm EDTA in PBS and lysed in RIPA buffer for further immunoblot analysis.

### Immunohistochemistry

Following removal of colonic contents, distal colonic sections were dissected and fixed in 10% formalin for 48 hrs. Sections were embedded in paraffin, sliced and stained with pNF-κB or Claudin-1, 2, 3 or 7 as previously described^41^.

### Statistical analysis

Graphs and statistical analyses were performed using GraphPad software V6.0 (GraphPad Prism Software). Results between tests and controls were compared using the Student’s t test. P values less than 0.05 were considered statistically significant.

## Supplementary information

Supplementary files

## Data Availability

The data analyzed during the current study are available from the corresponding author on reasonable request.
