# Supplementary material for: Cocaine Induces Inflammatory Gut Milieu by Compromising the Mucosal Barrier Integrity and Altering the Gut Microbiota Colonization
Source: Sci Rep. 2019 Aug 21;9:12187. doi: 10.1038/s41598-019-48428-2 (PMC6704112; doi:10.1038/s41598-019-48428-2)

Cocaine Induces Inflammatory Gut Milieu by Compromising the Mucosal Barrier Integrity and Altering the Gut Microbiota Colonization

Ernest T. Chivero[1], Rizwan Ahmad[2], Annadurai Thangaraj[1], Palsamy Periyasamy[1], Balawant Kumar[2], Elisa Kroeger[1], Dan Feng[1], Ming-Lei Guo[1], Sabita Roy[4], Punita Dhawan[2,3], Amar B Singh[2,3], Shilpa Buch[1*]

[1]Department of Pharmacology and Experimental Neuroscience, University of Nebraska Medical Center, Omaha, NE 68198, USA.
[2]Department of Biochemistry and Molecular Biology, University of Nebraska Medical Center, Omaha, NE 68198, USA.
[3]VA Nebraska Western Iowa Health Care System, Omaha, NE 68105, USA
[4]Department of Surgery, University of Miami, Florida, FL 33136, USA.

*Correspondence to Shilpa Buch: sbuch@unmc.edu, Tel: +1 402 559 3165.

Supplementary Table S1: Bacterial species altered by cocaine in the colon

Species	Fold change	P value	Significance	Regulation
Porphyromonadaceae unclassified	9.10	0.0041	yes	up
Bacteroidales unclassified	7.16	0.0015	yes	up
Barnesiella unclassified	10.65	0.0043	yes	up
Clostridiales Family XIII. Incertae Sedis unclassified	1.36	0.0051	yes	up
Erysipelotrichaceae unclassified	1.84	0.0065	yes	up
Sphingomonas unclassified	4.42	0.0020	yes	up
Pasteurellaceae unclassified	4.36	0.0130	yes	up
Proteobacteria unclassified	6.71	0.0166	yes	up
Mucispirillum unclassified	-1.34	0.0191	yes	down
Desulfovibrionaceae unclassified	4.53	0.0272	yes	up
Alistipes unclassified	2.25	0.0413	yes	up
Butyricicoccus unclassified	-0.88	0.0479	no	down
Uncultured Staphylococcus sp.	4.17	0.0451	yes	up
Ruminococcaceae unclassified	-0.51	0.0191	yes	down
Prevotella uncultured	inf	0.0130	yes	up

Supplementary Table S2: Bacterial species altered by cocaine in fecal droppings

Species	Fold change	P value	Significance	Regulation
Porphyromonadaceae unclassified	10.74	0.0015	yes	up
Barnesiella unclassified	Inf	0.0052	yes	up
Proteobacteria unclassified	7.18	0.0406	yes	up
Turicibacter unclassified	0.48	0.0415	yes	up
Bacteroides salyersiae	-1.20	0.0231	yes	down
Alistipes unclassified	2.68	0.0233	yes	up
Pseudoflavonifractor unclassified	-1.07	0.0281	yes	down
Bacteroides stercorirosoris	-1.55	0.0340	yes	down
Lachnospiracea incertae sedis unclassif	-1.25	0.0412	yes	down
Odoribacter unclassified	11.67	0.0360	yes	up
Streptophyta unclassified	-3.95	0.0360	yes	down
Uncultured *Lactobacillus*_sp.	-2.03	0.0497	yes	down

Supplementary Figure 1 Buch

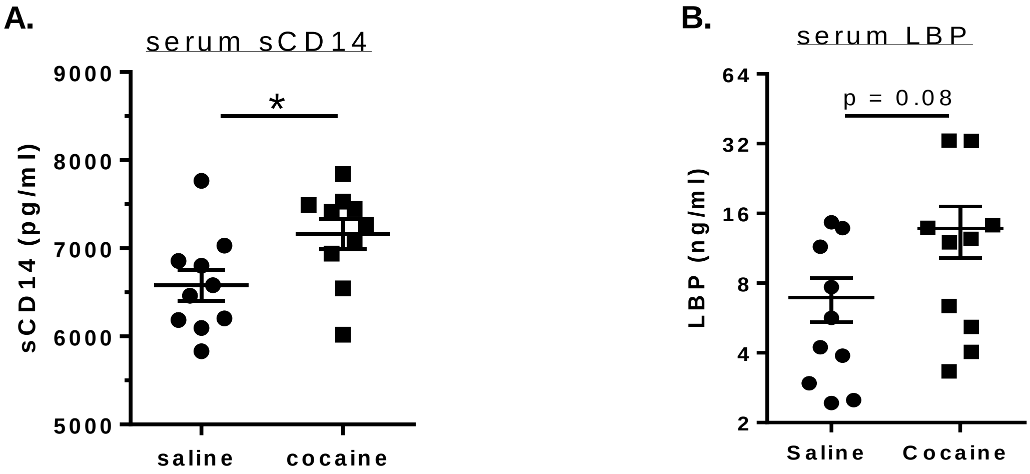

Supplementary Figure 1: Cocaine-induced an inflammatory milieu in the serum

Soluble CD14 (sCD14) and Lipopolysaccharide Binding Protein (LBP) were quantified by ELISA in serum samples from cocaine-administered mice or saline controls (n=10/group, *$p < 0.05$). As shown in a, sCD14 levels were increased in the cocaine administered group, and LBP showed an increasing trend (b) compared with the saline controls.

Supplemental Figure 3. Raw Western Blots for Figure 3

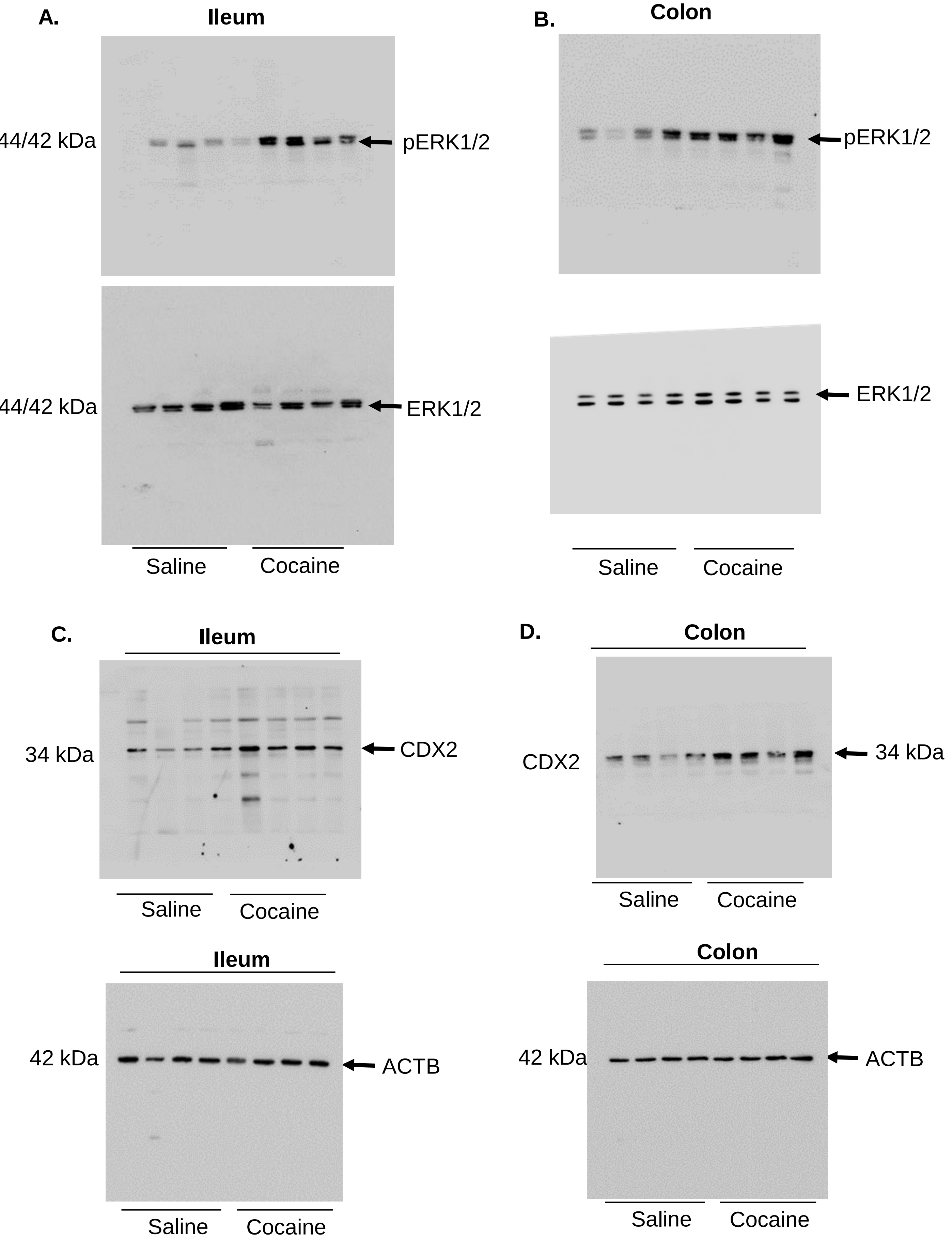

Supplemental Figure 4 . Raw Western Blots for Figure 4

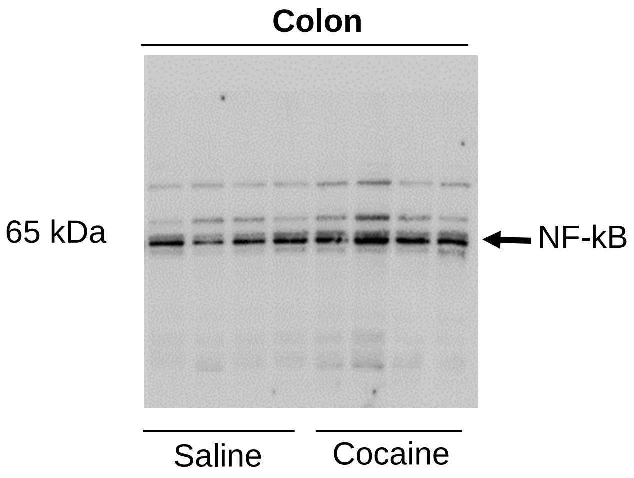

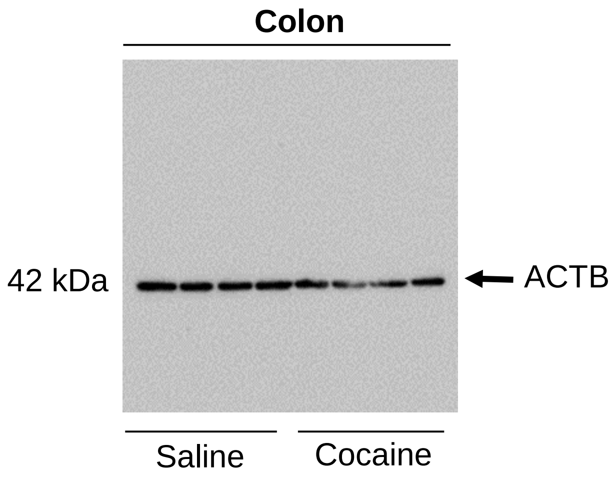

Supplemental Figure 6. Raw Western Blots for Figure 6

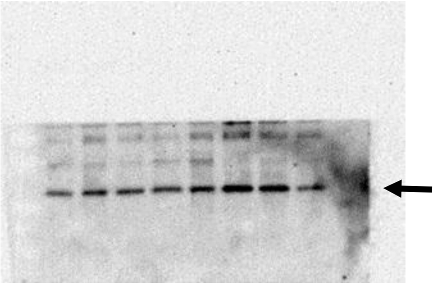

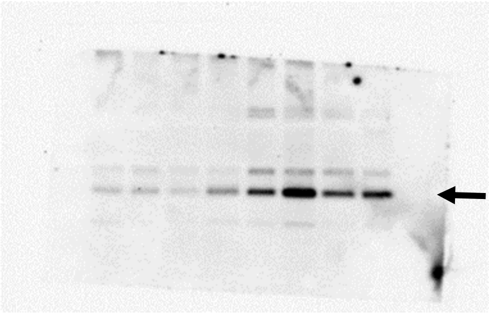

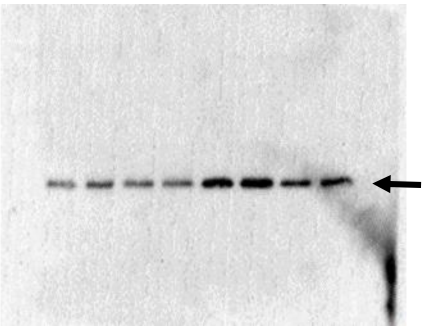

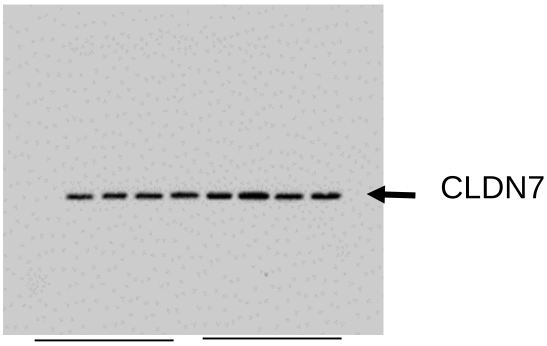

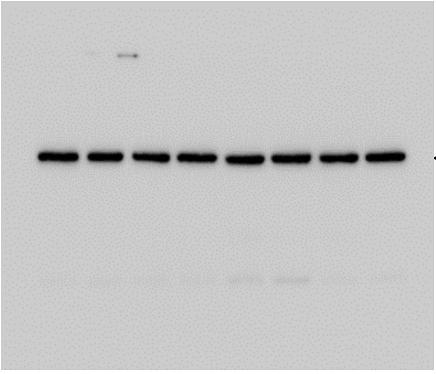

Supplemental Figure 7. Raw Western Blots for Figure 7

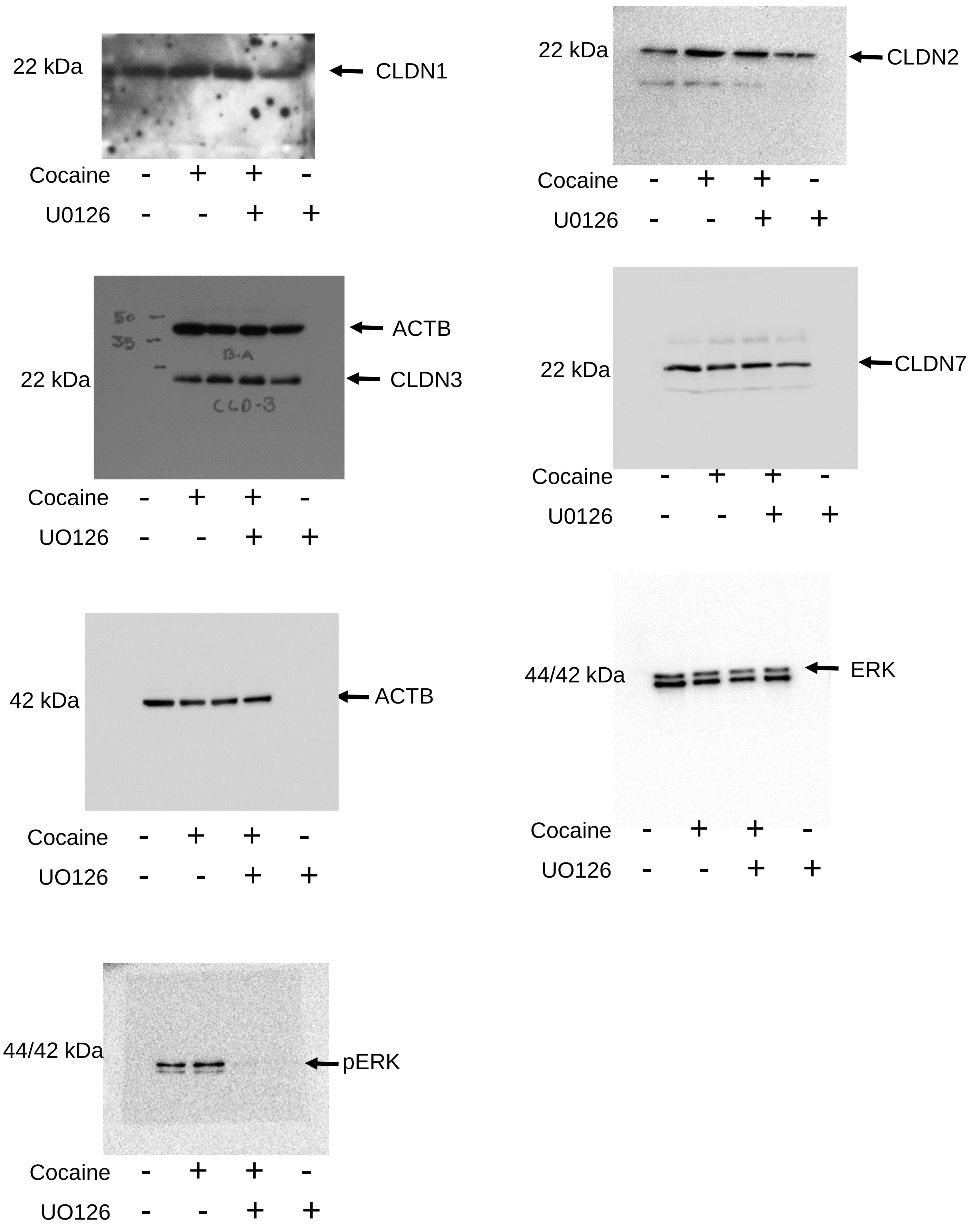

Supplement: Supplementary file 1 — Supplementary files [file 41598_2019_48428_MOESM1_ESM.pdf]
